# Cycles of judicial and executive power in irregular migration

**DOI:** 10.1186/s40878-017-0059-x

**Published:** 2017-10-11

**Authors:** Marinella Marmo, Maria Giannacopoulos

**Affiliations:** Flinders Law, College of Business Government and Law, Flinders University, Sturt Road Bedford Park, Adelaide, Australia

**Keywords:** Irregular migration, Executive power, Judicial power, Italy, Australia

## Abstract

This article argues that power struggles between judiciaries and executives are fuelled by tensions of securitisation, border control and human rights over the issue of irregular migration. The article juxtaposes three paradigm court cases to render the argument concrete, focusing on two Australian High Court decisions (*M70 v Minister for Immigration and Citizenship* and *CPCF v. Minister for Immigration and Border Protection & Anor*) and one decision from the European Court of Human Rights (*Hirsi Jamaa and Others v. Italy*). An examination of these cases reveals each step of this cycle: the executive attempts to produce a buffer to avoid or minimise migrants’ protections and judicial review, yet such manoeuvring is countered by the judges. Following this, new steps of the cycle occur: governments display disappointment to courts’ interventions in an effort to discredit the exercise of judicial power while the judiciaries maintain the focus on the rule of law. And so the cycle continues. The key argument of this paper rests on the paradox resulting from the executive’s attempts to curb judicial intervention, because such attempts actually empower judiciaries. Comparing different jurisdictions highlights how this cyclical power struggle is a defining element between these two arms of power across distinct legal-geographical boundaries. By tracing this development in Australia and in Europe, this article demonstrates that the argument has global significance.

## Introduction

The reconfiguration of executive powers in the area of border control and securitisation has been a central theme discussed in recent literature on mobility and border crossing (among others, Aas & Bosworth, 2013; Zedner, 2013; Mitsilegas, 2015a). Such discussion rotates around the concept of ‘crimmigration’ (Stumpf, 2006), referring to the employment of criminal law and security enforcement strategies in migration matters. Not only do executives produce new stringent rules on crimmigration, but such rules are implemented in their immediacy, freezing the time-space continuum to minimise or postpone parliamentary debate and judicial scrutiny (Giannacopoulos, Marmo, & De Lint, 2013). Such reconfigurations of power have had an impact on national legal frameworks with the effect of challenging international human rights principles. This has been theorised by Mitsilegas (2015b) as the emergence of the ‘law of the border’, which tests the ‘borders of law’ meaning the rule of law. Therefore the intervention of judiciaries in response to new configurations of power in crimmigration matters have been at the centre of academic debate (Legomsky, 2007; Aliverti, 2013; Aliverti & Bosworth, 2017). This article is concerned with the cyclical relationship between executives and the judiciaries on migration and their struggle for power. This cyclical relationship impacts on irregular migrants’ access to justice and human rights and so it is of central importance.

The analysis of the cyclical power struggle between these two institutions is based on three paradigm legal cases from Australia and Southern Europe: the *M70 v Minister for Immigration and Citizenship* (2011) (hereafter *M70*), the *CPCF v. Minister for Immigration and Border Protection & Anor* (2015) case (hereafter *CPCF*) and the *Hirsi Jamaa and Others v. Italy* (2012), (hereafter *Hirsi*). An examination of these cases allows us to offer a fresh perspective to migration studies on the significance of the cyclical relationship of the executive and the judiciary has in irregular migration.^1^ It adds to knowledge on complex migration politics across the two geo-political dimensions and it contributes to the discussion on the emergence of “judicial globalisation” (Slaughter, 2004, p. 67) as a countering force to new configurations of powers centred on border securitisation policies.

Methodologically this is a textually based critical analysis and comparison of legislation, migration policies, judgements and executive responses in Southern Europe and Australia. While comparative work on law questions is often undertaken across legal systems that share similar foundations and/or legal and language regimes (Giannacopoulos, 2016, p. 2) the approach we take here requires a break from this familiar approach. This article draws on primary legal texts as the basis for analysis but does not conduct a formal ‘black-letter’ legal analysis. It does however work in line with Hollifield’s insight that “in order to understand the politics of international migration we must compare immigration politics and policy outcomes among the liberal democracies” (Hollifield, 1992, p. 590). We focus ﻿here﻿ on the domestic structure of the judiciary and the internal domestic response of executives to judicial action on the international and complex question of migration. Since the objective here is to track a legal relationship that is of increasing relevance for migration, that of the executive and the judiciary, across two regions that are conventionally legally incomparable, a diverse range of texts have been brought into dialogue. Like Borkert and Penninx, we take an approach that stems from the idea that the multi-level governance of migration “is a process and not an event” and as such “is often of a cyclical in nature” (Borkert & Penninx, 2011, pp. 10–11). Borkert and Penninx identify the crucial importance of analyzing the role of various actors within these cyclical relations, even if comparative study in the field of migration is “by no means straightforward” (Borkert & Penninx, 2011, pp. 11–12). This is a complex study grounded in legal decisions with a focus on the relationships between actors and institutions (specifically the executive and judiciary) and their cyclical connections which assists in better understanding the contemporary trends of securitisation, extra-territorialisation and the undoing of protections for irregular migrants. Tracking this cycle reveals a transformation of the relationship itself as well as showing the profound effects this has on the lives of the peoples whose movements activate the governance cycle we track. This power contest is paradoxical in some ways because even if the executive attempts to curb judicial power that gesture also re-affirms the judiciary’s ongoing role in irregular migration matters.

The article is comprised of four interlocking parts that allow for this argument to be built and for it to unfold. In the first section, Becker’s hierarchy of credibility is explained as a theoretical framework through which the relationship between the executive and the judiciary can be seen. The second part briefly introduces the selected migration legal cases and begins to track the relevant parallels between the two distinct jurisdictions with which we are concerned. The third part discusses emerging power configurations, where immediate and stringent executive responses to mobility 'crises' aim at the reinforcement of a maritime buffer to contain and push back urgent threats. Part four tracks how the executive-judiciary cycle occurs in the selected court cases.

## The executive-judicial relationship and the hierarchy of credibility

Becker's (1967) ‘hierarchy of credibility’ model suggests that in each society "members of the highest group have the right to define the way things are" (Becker, 1967, p. 241). Becker theorises that the community is morally bound to accept the definitions and explanations provided by the highest group. ‘Truth’ is central to Becker’s analysis whereby truth from above is to be considered unquestionable. But when the community finds itself at odds with the explanations given by the highly ranked group, "officials develop ways of both denying their failure … and explaining those failures that cannot be hidden" (p. 243).

This theoretical framework explains the contradictory practices of executives where they are at once tough on borders but also immersed heavily in human rights protection talk. The production of buffers between irregular migrants and the destination country – via offshoring, push back policies, people swapping deals and 'saving lives' rhetoric – are acts that produce invisibility and allow governments to create narratives to dictate events as ‘true’. All other versions, offered by media, NGOs and, saliently to this paper, by the judiciary, are considered questionable because the executive considers itself as the highest ranked group and the ultimate truth-holder. By making the source of information, in this case the irregular migrants, difficult to reach through the amplification of buffers, all other agencies and bodies are discredited by the government, as they cannot access the source and therefore are represented as not fully ‘understanding’ the issue at hand.

Alongside the development of buffers and secrecy the increased militarisation of borders and the silencing practices engaged in by the executive are also paradoxically caught in a web of high visibility and spectacle. The executive’s efforts to achieve invisibility while displaying sovereign enforcement, control, and power creates its own spotlight (De Genova, 2013). Michael Welch (2012) has conceptualised this dynamic not only as visual but sonic. He argues that alongside the ‘quiet manoeuvring by the state’ there is the presence of ‘loud panic’ (p. 324). Ironically the silencing and shadowing of border practices become prominent subject matter in national debates.

Further counteracting the executive’s expansion of power through these border practices is the judiciary, which claims a rule-of-law stand on these matters the effect of which is to protect the migrants’ eroded human rights. Becker (1967, p. 243) would say that in overly charged matters, such as migration and border security, it is more complicated to understand: ‘who has a right to define’, and this becomes a ‘matter of arguments’. Both sides, the executive and the judiciary, claim the space of the ‘lawful’ and the protection of communities. Both sides therefore claim a moral stand, as per Becker’s terminology.

Becker (1967) has claimed that: "credibility and the right to be heard are differently distributed through the ranks of the system" (p. 241). The executive has cut out for itself a role ‘above’ the other branches of the state in matters of security and border protection. Reactions to the *M70*, *Hirsi* and *CPCF* judgements, discussed below, would suggest that the executive is imposing itself at the top of the hierarchical ordering for the protection of territory and people. The executive therefore claims the position of the highest ranked group in these matters that comes with the ‘benefit’ of defining facts and events as true.

Further, Becker (1967) identified the state’s capacity to deny responsibility by discrediting those who take the view of the powerless. When courts scrutinise matters other than those selected by the executive as having ‘sovereign importance’, it is expected that they take the position of deference to the executive will. Dauvergne (2008) refers to courts’ deference to executive will in US migration matters as a form of tradition (p. 47), signalling the positioning of the judicial body as a governance network to advance hegemonic power. Jamieson and McEvoy (2005) argue that the judiciary can be complicit in perpetuating the aims of the executive. Grewcock (2012) points out that the ‘evasion of responsibility’ by the state is allowed by ‘a degree of willing compliance, passive acquiescence and ingrained dependence’ present in the wider society and other state institutions (p. 55). Therefore, the formation of migration management regimes does not occur without the complicity of other high level of institutions.

Despite this scholars have identified the judiciary as having the capacity and willingness to dispute executive power and to produce an alternative narrative to counteract the executive action of the government in migration matters (Pickering and Weber 2012; Mitsilegas, 2015b; Aliverti & Bosworth, 2017). It is here that the judiciary may constrain state control on migration to service ‘higher aspirations, namely, the recognition of human rights and fundamental freedoms of all persons’ (Opeskin, 2012, p. 553). Slaughter (2004) identified the capacity of judiciaries to rise to the challenge of prioritising the enforcement of human rights in domestic courts as a result of a global judicial dialogue. Knowledge transfer among the transgovernmental judicial body is of fundamental value to furthering such an objective (Marmo, 2005; Marmo, 2012). Although the judiciary are represented as less credible in migration matters by the executive, this should not lead to the conclusion that judiciaries are without credibility and authority. After all, the area of refugee law, “has evolved mostly under the influence of judges” (Lambert, 2010, p. 4) which means that judges at the top of their judicial pyramid have been key agents of change in the refugee rights realm.

And yet, in the application of Becker’s hierarchy of credibility, each alternative view expressed by the judiciary is represented by the executive as controversial and often disguised as a matter of efficiency. By diluting, minimising, infantilising and denying the legitimate contribution of judiciaries to migration debates, executives apply Becker’s credibility concept, where the highest group is the only institution able to define ‘the way things really are’ (Becker, 1967, p. 241). This paper draws critical attention to these minimisation ﻿and 'truth' making practices.

This paper also demonstrates that for each executive’s attempt to discredit the judiciary or bypass it by introducing a new rule circumventing access to justice, the judiciary responds and reacts. It is in the continuum of countering by both sides that the cyclical power struggle is revealed in the realm of irregular migration governance. These divisions and tensions evident in liberal democracies do not actually weaken the role of judiciaries; their role is reinforced in cyclical ways as a key constitutive element of state power affecting the operations of sovereign control at borders. The cyclical relation we track here between executives and judiciaries plays out in this refugee law area where there is “tremendous opportunity in terms of seeking a greater transnational judicial role” (Lambert, 2010, p. 4). Although executives and judiciaries in the area of migration appear in a state of constant tension, it is this cyclical movement between them that allows their respective powers to meet and intersect to generate policing practices at borders and regimes of inclusion/exclusion.

## M70, Hirsi and CPCF: Parallels across jurisdictions

The socio-legal and political backgrounds of Australia and southern Mediterranean European Union (EU) members, such as Italy, are markedly different; yet, these liberal democratic countries are experiencing similar patterns of irregular migration, as the selected three key legal decisions reveal. We signal here that the Southern European Governments lie in a hierarchy of multilevel governance, both from the point of view of the legislation and of the judiciary, a regional layer that is missed in the Australian context. This structural difference however does not negate the importance or ability to stage a comparison of the continuities of logic that hold across the two zones. In fact it adds to knowledge on complex migration politics across the liberal democracies in question and it contributes to the analysis of the cyclical power struggle between the executive and judiciary as a common phenomenon that crosses geo-political boundaries. By comparing the cases *M70* in Australia and the *Hirsi* case in Europe, the striking similarities between actions of executives and reactions of courts are revealed as a cyclical power struggle.

The High Court of Australia handed down its *M70* decision in August 2011 and in that judgement declared Malaysia invalid as a destination for asylum processing despite the Minister for Immigration and Citizenship’s declaration. Unless the proposed processing country is bound by international or domestic laws to uphold basic human rights protections towards asylum seekers, then it could not be validly declared a processing country by the Australian Government according to the High Court of Australia.

The ECHR *Hirsi* decision came in February 2012 and was prompted by the Italian state’s attempt to re-direct populations in order to prevent arrival in Italy. The applicants were intercepted by the Italian military and returned to Libya where they were handed over to Libyan authorities. The Court found that Italy could not avoid responsibility as the applicants were in its territorial jurisdiction. But further to this, the state could not rely on the bilateral arrangement it had entered into with Libya as this would expose the asylum seekers to precisely the dangers that the Geneva Convention is in place to prevent.

These cases represent a historical juncture when the judiciary of each regional area had opportunity to examine similar national regimes of migration built on bilateral agreements with non-signatory countries (Italy-Libya and Australia-Malaysia). And, in each case the courts expressed similar views *against* those regimes (Kritzman-Amir & Spijkerboer, 2013). In other words in both those cases, each state was faced with a judicial response that curbed its executive powers. Kritzman-Amir and Spijkerboer (2013) have highlighted the ways that states like Australia and Italy have attempted to mitigate the tension that comes from a nation’s international law obligations and the deep desire to continue to remain sovereign. The ‘people swap’ and ‘push back’ strategies in the context of *M70* and *Hirsi* were two such attempts. Den Heijer (2013) has noted that the High Court of Australia ‘employed fairly similar considerations’ to those used in the *Hirsi* case in highlighting that the Malaysia ‘refugee swap’ was contravening international rules on non-refoulement (p. 280). Tondini (2012) referred to the ‘striking similarity’ between the two bilateral agreements ‘with regard to the absence of a functioning asylum system’ both in Malaysia and Libya (p. 69).

The 2015 *CPCF* decision provides further nuance to the analysis of cyclical power. This Australian case also pivots around interception at sea and the push back of non-nationals. The *Hirsi* and the *CPCF* events both took place in non-territorial waters in a normalised routine of interception and rejection. In these instances, the respective states’ ‘border protection’ military personnel took on board undocumented people without regard for identification processes. In both *Hirsi* and *CPCF* asylum seekers were detained using military state powers, but the length of detainment varied significantly from 10 h in *Hirsi* to 1 month in *CPCF*. The Australian judges did state that the threshold of safety, closely linked to the principle of non-refoulement, is a requirement of Australian laws but with a close 4–3 judgement, concluded in favour of the Australian government, claiming the executive acted within the parameters of the *Maritime Powers Act* (2013), passed by the Australian government after the *M70* judgement. As distinct from *Hirsi* and *M70* with the *CPCF* case, the High Court of Australia held that detainment at sea for the purpose of preventing arrival of unauthorised migrants to Australia and push back to safe port was lawful.

While the *CPCF* development does not allow the claim that executives and judiciaries are pulling in opposite directions in their attempts to impact the refugee realm, it does add depth to the discussion of the cyclical power struggle. The decision of the High Court came in January 2015. While the judges were considering the *CPCF* case, the Australian government passed a number of amendments to the *Migration Act* and to the *Maritime Powers Act* in December 2014 to allow for the removal from high seas of unauthorised migrants without breaking national laws. The *non-refoulement* principle, emanating from the international realm but having domestic relevance, is the thread that links these judicial cases and is precisely the refugee protection at stake in this discussion. This principle has been established in the 1951 Convention Relating to the Status of Refugees (otherwise known as the Geneva Convention) and has acted as arguably the most significant protection since that time. We make this point not as hyperbole but to highlight the fundamental idea that it is the right to be kept away from and not returned to places of danger that gives meaning to the concepts of asylum and refuge. However, while this principle is part of the EU legislation and also binding to Australia, the section below addresses how it has been undermined at an executive level through a securitisation agenda.

## Emerging power configurations and reduced access to justice

Despite the magnitude of the irregular migration issue in the southern Mediterranean region being fifteen times the size of the Australian challenge in the period 2006–2013 (Parliament of Australia, 2015; UNCHR, 2015), the executive responses in each country highlight parallel approaches. In a key text in this area, Gammeltoft-Hansen (2011) examines the problematic (from a refugee protection standpoint) policy developments of ‘extra-territorialisation’ or ‘offshoring’ which may involve the interception of migrants on the high seas, extra-territorial processing of refugees or the excision of territory to evade jurisdiction and legal obligations (see also De Boer, 2015, p. 2). These strategies, which also have a militaristic flavour, have emerged as significant trends in both Australia and Southern Europe in the search for policy solutions. Hyndman and Mountz (2008) argue that the externalisation of asylum and migration refers to the ‘strategic geographical tactics’ (p. 249) used by states to create buffers between the origin and destination countries. This strategy prevents migrants from joining ‘the juridical order’ (p. 259) meaning they are unable to have their case heard by destination country domestic courts. In Australia these trends emerged strongly in 2001 after the Tampa incident, in which mostly Afghani asylum seekers attempted to arrive in Australia (Giannacopoulos, 2014a). The shift away from an international law protection approach was clearly signalled by the insistence that asylum seekers be denied entry to mainland Australia. This acted as the impetus for a range of retrospective legislation that would lead to the regionally based Pacific Solution (Giannacopoulos et al., 2013) followed by other similar agreements under the following governments (Grewcock, 2014). In the European context, in which Southern member states are seen as the ‘gateway’ to Europe by those from North Africa who seek entry to the EU via the Mediterranean (Kneebone, McDowell, & Morrell, 2006), Greece and Italy have also been described as the ‘outposts’, charged with the burden of ‘fencing off’ (Triandafyllidou & Ambrosini, 2011; Triandafyllidou, 2014) access to the EU. In response to this pressure, Italy has entered numerous bilateral agreements with Libya (Giuffré, 2012) which has led to ‘push backs’ from Italy to Libya after interception at sea. This paralleled Australia’s move towards regional agreements, such as the 2011 Malaysia ‘refugee swap’ solution which was the plan to deport 800 future irregular maritime arrivals in Australia to Malaysia in exchange for 4000 United Nations High Commissioner for Refugees (UNHCR) recognised refugees over the following 4 years (Kritzman-Amir & Spijkerboer, 2013, p. 12).

In both regional areas the attempt to ‘rescale governance’ and manoeuvres of ‘political mobilization’ aim at producing a ‘reconfiguration of sovereignty through regional and national management regimes’ (Loyd & Mountz, 2014, pp. 24–25). Management regimes take place in what Garland (2001) refers to as ‘the culture of control’, a punitive law-and-order approach that reshapes the thinking process of globalisation and justice. The rescaling of governance ensures defensive mechanisms are proposed by the state under the pretext of border protection (Aas & Bosworth, 2013; Zedner, 2013) so that a climate of ‘emergency’ is produced. Gammeltoft-Hansen (2010) argues that sovereignty has been commercialised and can be an object of trade. Irregular migration policies therefore give rise to new forms of legal governance that increase state powers (Giannacopoulos et al., 2013). These emerging power configurations are enforced in their immediacy meaning that opportunities for scrutiny by institutions like the judiciary are minimised. Critical assessment of how state practices function to reduce accountability is required in liberal democracies where executive power is expanding. And yet there is an absence of scrutiny and necessary tools to fend off mobility emergencies. This process is not new, but is becoming more significant to political and legal authorities as they attempt to broker competitive advantages in conditions of globalisation whilst postponing or avoiding scrutiny by domestic and international bodies.

Maritime or water borders are a geographical commonality for Australia and Southern Europe contributing to similar challenges in the fluidity of power-shifting mechanisms, due to both increased visibility and invisibility. The high seas become sites for maritime drama or ‘loud panic’ (Welch, 2012, p. 325) even as they are spaces that cannot be fully scrutinised. The maritime zones are the spaces in which the militarised responses to asylum play out, contributing to conditions that allow information to be monopolised and cultivated politically. Water borders also allow for expanding ‘othering strategies’ (Jamieson & McEvoy, 2005), which function to displace international human rights protocols by constructing asylum seekers as undeserving of protection. By entering into immediately enforceable bilateral agreements with non-signatory neighbouring nations, Italy and Australia are actively engaged in shrinking the space for human rights protections available for asylum seekers. The privatisation process of ‘migration containment’ on offshore detention centres creates a buffer between the state and international law norms. Removing or diluting responsibility of the state by the formation of contractual relations allows the state to produce a ‘truth’ of events without compromising its positioning in the global human rights agenda. Underwriting all of these trends is the geopolitical reality that water borders either in Australia or Southern Europe delineate the very space of socio-economic and political divisions. Pugliese argues that in the case of Italy, the prison island of Lampedusa represents ‘the fault line between Europe and Africa’ whereas ‘Christmas Island marks the fault line between Australia and Asia’ (Pugliese, 2010, p. 117). While it is arguable whether Christmas Island and Lampedusa form part of Australia and Italy, these zones are significant to migration questions precisely because they act as internal/external zones. The ‘symbolic’ and the ‘legal’ perspectives of those two islands are contradictory and pull their narrative in opposite directions. The symbolic narrative used in migration discourse has been for non-inclusion of asylum seekers into the national order and so has functioned as the pre-cursor to the broader attempts to externalise asylum even when legally tracked in those islands. We argue that the effect of the symbolic narrative in both zones is to keep the economic, social and racial divisions intact between the first and third worlds while also silencing alternative ‘truths’.

The reinforcement of a buffer has been addressed in Australia through continued *offshoring* and through more intense militarisation in the guise of *Operation Sovereign Borders.* This conservative coalition party’s operation was introduced by the Australian Government under Prime Minister Abbott in 2013 (Australian Government, 2013). This was a military-led response aimed to decrease entry of asylum seekers to Australia by redirecting those people to neighbouring islands. In 2017, the Minister for Immigration and Border Protection Peter Dutton boasted that there have been ‘1000 days since the last people smuggling boat reached Australia and more than 3 years since the last known death at sea at the hands of people smugglers’ (Dutton, 2017), engaging in the double act of security policy the enactment of securitisation alongside human rights protection rhetoric. With increasingly minimal information shared by the Australian government in the formation-process of a ‘on-water-matters’ secretive regime, Australia has been harshly criticised (among others, UNHR, 2016).

Italy recently attempted but ultimately abandoned a more humane effort in managing people movements from North Africa. The Italian *Operation Mare Nostrum* began in October 2013 with the aim of intercepting people in distress and carrying them back to Italian reception centres (Mare Nostrum, no date). Because of its emphasis on rescuing and assisting, the operation *Mare Nostrum* received strong support from intergovernmental agencies such as the International Organisation for Migration but no support from other EU member states with the UK harshly criticising the operation as being a pull factor (Travis, 2014). While Italy was seeking support from the EU to continue the Operation, according to Amnesty International ‘the EU and its member states avoided any decisions which could help refugees and migrants leaving North Africa for as long as they could’ (Amnesty International, 2015, p. 34). On 27 August 2014, Operation Triton, an EU border patrol-only operation managed by Frontex, the European border agency, effectively replaced the Operation *Mare Nostrum* (European Commission, 2014). Operation Triton only monitors the ‘external border’ (European Commission, 2014), up to 30 nautical miles from the European coasts, and is not a rescuing migrants at sea but ‘render[s] assistance to persons in distress’ (European Commission, 2014). Centred on these points, Operation Triton was criticised as it was evident it resumed the harsher approach (“Frontex Launches Joint Operation Triton”, 2014). More recent tragedies on the Mediterranean have prompted the UN High Commissioner for Refugees Antonio Guterres to say that Triton is a ‘woefully inadequate replacement for Italy's Mare Nostrum’ (“Hundreds of Migrants”, 2015, para. 4).

## Executive prerogative under judicial scrutiny: The cyclical power struggle

The arrival by sea of unauthorised, undocumented people has been increasingly described as a matter of national security, in which the rights of the nation state are being encroached (Long, 2009). While governmental powers move to prohibit irregular migration, decisions of the highest courts in Australia and Europe have indicated an attempt to limit such executive power by invoking the relevance of international law (Hyndman & Mountz, 2008; Mitsilegas, 2015b). Therefore, as states introduce more legislation to criminalise irregular migration and build legislative walls to limit judicial intervention (Pickering, 2005; Kneebone, 2009; Ng, 2012), we argue that the judicial function in adjudicating on these matters is, paradoxically, reinforced. Even if the executive has attempted to minimise the importance of the courts in matters of national sovereignty (Giannacopoulos et al., 2013), courts continue to play a significant role in this realm. The power struggle between the executive and the judiciary is apparent when the judiciary is characterised by leading political figures as undermining legitimate, legislatively based executive policy. *M70*, the decision to disallow the offshore processing arrangement (O’Sullivan, 2011), was described by the then Immigration Minister as ‘profoundly disappointing’ (*"Australia Court Rules Out Refugee ‘Swap’ With Malaysia",* 2011, para. 4). A similar sentiment was expressed by the former Italian Minister of the Interior, who declared that the decision reached in *Hirsi* rejecting comparable offshore processing was ‘incomprehensible’ (Polchi, 2012, para. 5). This power dynamic is not unique to these court cases. For example, similar approach can be found in the ECHR *M.S.S*. case in 2011, whereby the unjust and harsh treatment of asylum seekers in Greece, condemned by the European Court of Human Rights in 2011 was dismissed by Greek politicians who invoked the Greek cultural trait of ‘philoxenia’, or hospitality, as a way of nullifying criticism (Cheliotis, 2013). These developments reveal the executive and judiciary as key, yet conflicting institutions in the governance of irregular migration. They also reveal the key aspects of Becker’s hierarchical belief of ‘moral quality’, of those high in ranks, in this case the executive, which imposes definitions and explanations about facts and events on community members. This is especially the case in highly charged matters, where the executive is asserting dominance and expanding power. The judiciary, by responding and intervening – or ‘interfering’ – also claims a moral stand on these matters, after all they are protecting access to justice and upholding human rights. In the attempt, on both sides, to claim the space of the ‘lawful’, the cyclical power struggle in the realm of irregular migration governance is revealed. A closer look at the *M70*, the *CPCF* and the *Hirsi* judgements, in turn, can demonstrate these points further.

In the introduction of his judgement in *M70* Justice French identified the highly charged nature of this legal adjudication since ‘these proceedings involve legal issues which arise in a strongly contested area of public policy’ (*M70*, 2011, para. 1). He referred explicitly to a ‘public policy contest’ in regards to ‘the way in which Australia deals with non-citizens who enter its territory by sea without visas and invoke Australia’s protection obligations under the Convention relating to the Status of Refugees’ (para. 1). He gestured towards the inability of separating the work of the executive and the judiciary in the realm of asylum seeker governance while also claiming that the role of the judiciary is clearly defined. He stated:

Some decisions of this Court have had practical consequences for the implementation of government policy. It is the function of a court when asked to decide a matter which is within its jurisdiction to decide that matter according to law. The jurisdiction to determine the two applications presently before this Court authorises no more and requires no less (*M70*, 2011, para. 2).

This declaration on the ability to undertake purely legal work takes a narrow reading of ‘according to law’. While the judge sees his work in the courtroom as ‘law’ work, the work of the government to extend its powers is also legally permissible.

The applicants in *M70* were both citizens of Afghanistan who arrived at Christmas Island on 4 August 2011 in a boat that had sailed to Australia from Indonesia. The applicants both claimed that ‘they had a well-founded fear of persecution in Afghanistan’ (*M70*, 2011, para. 3). The fear of persecution is a required trait for someone seeking recognition as a refugee according to the Refugee Convention. Since there is no absolute right to asylum but only a right to seek it, the principle of *non-refoulement* is considered an absolutely critical aspect of the Refugee Convention since it seeks to prevent the return of refugees to places of danger (O’Sullivan, 2014). This protection is not, strictly speaking, undermined if refugees are sent to third countries designated as ‘safe’. On 7 May 2011, a bilateral arrangement was entered into between the Prime Ministers of Australia and Malaysia meaning that asylum seekers arriving by sea would be transferred to Malaysia for assessment. In exchange, Australia would expand its humanitarian program in resettling refugees who were residing in Malaysia (*M70*, 2011, para. 8). This Solution aimed to dismantle the ‘people smugglers’ business model’ as per former Prime Minister Julia Gillard’s statement in May 2011 (Maiden, 2011, para. 20). The logic of this new and ‘good deal’ furthered the concept of ‘good refugee’ (McAdam, 2013, p. 437) who waits in the camp as distinct from her opposite: the ‘criminal’ and ‘undeserving’ ‘queue jumper’. Four years earlier in 2007 Italy and Libya signed a bilateral cooperation agreement ‘to combat clandestine immigration’ which according to Article 2 of the Agreement required both countries to undertake ‘surveillance, search and rescue operations’ in ‘the departure and transit areas of vessels used to transport clandestine immigrants’ (*Hirsi*, 2012, para. 19).

The bilateralism present in both the Australian and Italian cases shows an anti Geneva Convention deployment of the concept of *non-refoulement* since the underlying logic of the agreements was to redirect asylum seekers to third zones of questionable safety. The contest that played out in the judicial realm though was whether the countries like Malaysia and Libya could be deemed ‘safe’ third places. Justice French was didactic on this point:

The questions the Minister must ask himself about whether relevant “access” and “protection” are provided and “human rights standards” are met, are not questions that can be answered without reference to the domestic laws of the specified country, including its Constitution and statute laws, and the international legal obligations to which it has bound itself (*M70*, 2011, para. 66).

He further instructed the Government of the day:

The Minister must ask himself the questions required by the criteria on the assumption that the terms “provide” and “meet” require consideration of the extent to which the specified country adheres to those of its international obligations, constitutional guarantees and domestic statutes which are relevant to the criteria (*M70*, 2011, para. 67).

It is evident from this approach that despite the Australian judge wanting to appear strictly legal and non-political, his work is profoundly enmeshed in border politics. He drew on refugee convention principles to nullify the executive’s Agreement with Malaysia. In a last minute intervention the executive was stopped by the judiciary on the morning the first exchange of refugees was due to take place. The judgement of the High Court was that ‘the ministerial declaration of 25 July 2011 was affected by jurisdictional error. It was not a declaration authorised by s 198A of the Migration Act’ (para. 68).

The Immigration Minister responded with equal amounts of judgement towards the judiciary. By describing the ruling as ‘profoundly disappointing’ ("*Australia Court Rules Out Refugee ‘Swap’ With Malaysia",* 2011, para. 4), the Minister attempted to represent the judicial position as the act of a misbehaving child. The infantilisation of the judicial body and attempting to erode judicial credibility by ascribing illegitimacy to the Courts’ role, occurred because the judiciary’s work was seen to be countering state intentions (Opeskin, 2012). But in what can be seen as a response to the response, Chief Justice French remarked:

[j]udicial review is an inescapable feature of any society governed by the rule of law under a written constitution where the legislature and the executive have limited powers. Its application to sensitive areas of official decision-making can sometimes generate inconvenience and cost and elicit legislative responses. […] Importantly, judicial review […] is essentially on the sidelines of the important debates which determine the future direction of Australia's public policy in relation to migration (Chief Justice French, 2011, p. 27).

It would appear that the Prime Minister did not agree that this was a marginal or sideline public policy decision.

The *CPCF* case added another tassel to the cycle of power between the two institutions. In 2014, deep in the Indian Ocean and away from public view, one of Australia’s Commonwealth vessels detained unauthorised migrants for 1 month. And so began the executive’s move to reassert power at the border and beyond when 157 Tamil Sri Lankan people were intercepted on 29 June and were detained on the high seas until July 22 (*CPCF*, 2015, para. 1 and 3). Immigration Minister Scott Morrison said at the time: ‘they will never be resettled in Australia’ (Dias, 2014, para. 8). The legal manoeuvres engaged in by the executive at this time can be seen as an attempt to recoup its supremacy in migration governance following the dominance of the Court in *M70*.

When the High Court issued an injunction on July 7 to prevent the detained people being returned to Sri-Lankan authorities, it was clear that once again the executive and judiciary were locked in battle. The injunction forced the Immigration Minister to have to admit that the refugee vessel in question existed and had been intercepted. The people on the vessel had already been detained for a week until it was made public knowledge against the wish of the executive ("High Court injunction blocks handover", 2014).

As the detention in international water was taking place, the executive rushed through Parliament the *Migration and Maritime Powers Legislation Amendment (Resolving the Asylum Legacy Caseload) Bill 2014*, which continues to attest to the ongoing contest of power between the executive and the judiciary. The Bill sought to restrict the judiciary’s ability to provide ‘critical oversight’ by way of judicial review leaving ‘Australia’s refugee protection regime as a matter almost entirely for executive or Ministerial discretion’ (Australian Lawyers for Human Rights, 2014, p. 3). As Richard Marles MP stated:

There is much in this bill which is, in effect, a legislative response to actions of the judiciary… In essence, what this legislation seeks to do is to scuttle one High Court case… Were this schedule to be passed, it would make the role of the High Court redundant. In our view, that is inappropriate (Commonwealth of Australia. Record of Proceedings, October 22, 2014, pp. 11573–4).

And yet the *Migration and Maritime Powers Legislation Amendment (Resolving the Asylum Legacy Caseload) Act* (2014) was passed by both Houses in December 2014 over a month before the final judgement of *CPCF* was handed down on 28 January 2015. This Act unambiguously placed border policing ahead of asylum seeker rights as expressed in UN Conventions and is clear and unapologetic about doing so. While there is no space to undertake a full analysis of that legislation here, it is necessary to highlight some key sections. Section 75A clearly sets out that ‘failure to consider international obligations [..] does not invalidate exercise of powers’. Section 75B stipulates that ‘rules of natural justice do not apply to exercise of powers’. Section 75E states that ‘powers are not limited by the *Migration Act* (1958)’ and section 75F cements the central role of the Minister who ‘may give directions about exercise of powers’ (Migration and Maritime Powers Legislation Amendment (Resolving the Asylum Legacy Caseload) Act (2014)). It should come as no surprise that in the context of this highly politically charged climate, the final ruling in *CPCF* was that Australia could detain asylum seekers on the high seas. The High Court ruled that holding the Sri-Lankan asylum seekers at sea was indeed lawful (*CPCF*, 2015, para. 43–50; O’Sullivan, 2015). In the majority decision, Chief Justice French held that Section 72 (4) of the *Maritime Powers Act* does not contravene Australia’s international law obligation of *non-refoulement* (*CPCF*, 2015, para. 11), effectively implementing section 75A *Migration and Maritime Powers Legislation Amendment (Resolving the Asylum Legacy Caseload) Act* (2014).

*Hirsi* also reproduces the centrality of human rights as expressed through the choice to uphold the principle of *non-refoulement.* The:

Principle of *non-refoulement*, as interpreted by the ECHR, essentially means that states must refrain from returning a person (directly or indirectly) to a place where he or she could face a real risk of being subjected to torture or to inhuman or degrading treatment (*Hirsi*, 2012, para. 34).

While the Italian state was concerned with avoiding human rights responsibilities by engaging in ‘turning back’ the ‘group of around two hundred people who left Libya aboard three vessels with the aim of reaching the Italian coast’ (para. 9) the ECHR was unforgiving. The Court disallowed Italy to deny responsibility in attempting to *refoule* by asserting that a clear jurisdictional link is formed through a causal legal chain. In other words, the rescue process establishes the jurisdiction of the rescuing state over the non-nationals. In finding a jurisdictional link existed, the ECHR held the Italian state to account:

Whenever the State through its agents operating outside its territory exercises control and authority over an individual, and thus jurisdiction, the State is under an obligation under Article 1 to secure that individual the rights and freedoms under Section I of the Convention (*Hirsi*, 2012, para. 74).

Here the Court was dismantling the attempts of the Italian state to assert power in extraterritorial space without responsibility. By ruling that ‘a vessel sailing on the high seas is subject to the exclusive jurisdiction of the State of the flag it is flying’ (para. 77) the judges were unambiguous: ‘the interception and rescue on the high seas of persons in distress cannot be re-packaged as a maritime police operation’ since it is ‘an obligation imposed by international law … (the Montego Bay Convention)’ (para. 65). The ECHR disciplined the executive and curbed their attempts to assert power extraterritorially by disallowing ‘turn-backs’ to be re-written as ‘rescue’ operations (para. 79).

Following the Court’s pronouncements in *Hirsi,* leading political figures stepped up to comment on the ruling as undermining legitimate, legislatively based executive policy. The term ‘incomprehensible’ used by the then Immigration Minister of Italy to describe the judgement (Polchi, 2012, para. 5) repeats the strategy of undermining the credibility of the judiciary by representing the judicial body as incapable of fully grasping the complexities at stake. Here, disciplinary and security discourses imposed by the executive crystallise an ordering principle by diluting the legitimate contribution made by the judiciary to the narrative of individual protection. This suggests that when the executive has faced challenge, their response has been to infantilise the challenger in order to displace the substance of the claims being made. Without wanting to agree with the executive regarding the Court’s naivety, we want to signal that the dynamic between the executive and the judiciary cannot be reduced to the simple contest between sovereign power and human rights virtue. The ECHR cites the Committee for the Prevention of Torture report condemning Libya as an unsafe country ‘in terms of human rights and refugee law’ (*Hirsi*, 2012, para. 36). There are multiple contradictions structuring this simple representation. The rationales of both the *Hirsi* and *M70* judgments pivot around a simplistic binary that organises countries into good/bad, safe/unsafe from the perspective of the human rights framework of refugee protection. Libya and Malaysia were posited as bad/unsafe and based on this logic the executives were curbed by the Courts in extending their respective powers. But given that the executive/judicial tension arose because of the unwillingness/willingness to uphold human rights in countries ostensibly supportive of refugee protections it should not be readily assumed that safety is assured in the liberal democratic states.

## Concluding remarks: Sovereign developments

There is no doubt that the contemporary sovereign state has been caught in the dilemma of reconciling human rights commitments with desire to maintain postcolonial prerogatives to decide who can enter the receiving society (Marmo & Smith, 2012). This has led to an increased emphasis on national security responses with borders being increasingly securitised and militarised (Michalowski, 2007). We have illuminated this by focusing on the continuities between the developments in *M70, Hirsi* and *CPCF* to show how the live issue of irregular migration is contracting and expanding judicial and executive power in the realm of migration politics. This power cycle is operating to restrict the space for protections in irregular migration.

The political and legal terrain with which we have been concerned will continue to change and unravel and continues to transform before us in the latest iteration of Europe’s asylum crisis. The article argues that, despite clear tensions between the two institutions and although they attribute to each other different hierarchies of value in an application of Becker’s theoretical framework, the executive and judiciary are increasingly interlinked in reconfiguring the legalities of irregular migration policies. This circulation of power has been occurring because the executive’s actions to extend their own power has triggered and, in turn, affirmed the role of the Courts. Examined through this lens, the article claims that the judiciary will continue to have a significant role to play in migration matters because - not despite – the executive attempting to minimise the importance of the courts in matters of national sovereignty. As states introduce more legislation to criminalise irregular migration and build legislative walls to limit judicial intervention (Pickering, 2005; Kneebone, 2009; Ng, 2012), the judicial function in adjudicating on these matters will be paradoxically, reinforced rather than eroded.^2^

The acknowledgement made by the ECHR in *Hirsi* regarding the increased pressure experienced by nations with maritime borders especially in the context of the economic crisis (*Hirsi*, 2012, para. 122), is of great significance. It is economic hardship, caused by political, religious, cultural and financial factors that are largely responsible for the desire of vulnerable people to move, in turn triggering states’ desire to frustrate those moves. It is in this context that we have seen the judiciary cut down for seeking to enshrine protections for asylum seekers using human rights frameworks. ‘Exclusion from status’ in states like Australia and Italy and/or the EU are a ‘key mechanism through which migrant lives are regulated’ (Ziadah, 2016). Entitlement to migration ‘status’ is a legal and political question that is impacted profoundly by the relationship of executive and judicial power. We have in a small way responded to De Genova and Peutz’s call for ‘scholars, advocates, and activists- citizens, denizens and deportees alike- to engage politically and theoretically in renewed ways with questions of freedom, in one of its most basic and meaningful senses: the freedom to traverse space and to make a space for oneself in the world’ (De Genova & Peutz, 2010, p. 3). With ‘Italy facing a refugee crisis on a scale far beyond Australia’s imagining’ (King, 2016) it continues to be important to take global and interdisciplinary perspectives not only to migration issues but to the legal relationships that are shaping those very issues. Clearly there is much work remaining to be done around the paradoxes we have unravelled.

## Abbreviations

ECHR: European Court of Human Rights
EU: European Union
UNHCR: United Nations High Commissioner for Refugees

## Footnotes

1 ‘Irregular migration’ generally relates to ‘entry, stay or work in a country without the necessary authorization or documents required under immigration regulations’ (International Organisation for Migration, 2011, para. 17).

2 It is interesting to note that the new Italy-Libya agreement dated February 2017 has been suspended by the Libyan Supreme Court in March 2017. This is another executive-judicial power struggle to consider and certainly adds to our argument of cyclical power of governments and courts.
